# Long term complete response of advanced hepatocellular carcinoma to glypican-3 specific chimeric antigen receptor T-Cells plus sorafenib, a case report

**DOI:** 10.3389/fimmu.2022.963031

**Published:** 2022-08-17

**Authors:** Hongwei Sun, Chongyun Xing, Songfu Jiang, Kang Yu, Shengjie Dai, Hongru Kong, Yuepeng Jin, Yunfeng Shan, Wenjun Yang, Zhen Wang, Jun Xiao, Huamao Wang, Wei Wang, Zonghai Li, Keqing Shi

**Affiliations:** ^1^ Department of Oncology, The First Affiliated Hospital of Wenzhou Medical University, Wenzhou, China; ^2^ Department of Hematology, The First Affiliated Hospital of Wenzhou Medical University, Wenzhou, China; ^3^ CARsgen Therapeutics Ltd., Shanghai, China; ^4^ Translational Medicine Laboratory, The First Affiliated Hospital of Wenzhou Medical University, Wenzhou, China

**Keywords:** chimeric antigen receptor T cell therapy, liver cancer, hepatocellular carcinoma, glypican-3, sorafenib

## Abstract

The clinical efficacy of current therapies for Hepatocellular carcinoma (HCC) are unsatisfactory. In recent years, chimeric antigen receptor (CAR) T-cell therapies have been developed for solid tumors including advanced HCC (aHCC), but limited progress has been made. Glypican-3 is a promising immunotherapeutic target for HCC since it is specifically highly expressed in HCC. A previous study indicated that GPC3-targeted CAR T-(CAR-GPC3) cells were well-tolerated and had prolonged survival for HCC patients and that Sorafenib could increase the antitumor activities of CAR-GPC3 T-cells against HCC in mouse models. Here, we report a patient with aHCC who achieved a complete response (CR) and a long survival period after the combination therapy of CAR-GPC3 T-cell plus sorafenib.

A 60-year-old Asian male diagnosed with hepatitis B virus (HBV) related HCC developed liver recurrence and lung metastasis after liver tumor resection and trans-arterial chemoembolization therapy. The patient also previously received microwave ablation therapy for lung metastasis. After the enrollment, the patient underwent leukapheresis for CAR-GPC3 T-cells manufacturing. Seven days after leukapheresis, the patient started to receive 400 mg of Sorafenib twice daily. The patient received 4 cycles of CAR-GPC3 T cells (CT011) treatment and each cycle was divided into two infusions. Prior to each cycle of CT011 treatment, lymphodepletion was performed. The lymphodepletion regimen was cyclophosphamide 500 mg/m^2^/day for 2 to 3 days, and fludarabine 20-25 mg/m^2^/day for 3 to 4 days. A total of 4×10^9^ CAR-GPC3 T cells were infused. The CT011 plus Sorafenib combination therapy was well tolerated. All the ≥ grade 3 AEs were hematological toxicities which were deemed an expected event caused by the preconditioning regimen. This patient obtained partial responses from the 3^rd^ month and achieved CR in the 12^th^ month after the first cycle of CT011 infusion according to the RECIST1.1 assessment. The tumor had no progression for more than 36 months and maintained the CR status for more than 24 months after the first infusion.

## Introduction

Hepatocellular carcinoma (HCC) is the most common histologic subtype of primary liver cancer, which is the sixth most common cancer and the third leading cause of cancer-related death worldwide (1). A combination of atezolizumab, an immune checkpoint inhibitor, plus bevacizumab is the current preferred first-line regimen (2). However, the objective response rate (ORR) of 27.3% is unsatisfactory in patients with unresectable HCC (3). The approval of atezolizumab plus bevacizumab suggests that immunotherapy combined with other medication is an effective therapeutic strategy to achieve a better clinical response. The combination of immunotherapeutic agents with other medications, including tyrosine kinase inhibitors, has also been demonstrated to be effective as first-line in patients with metastatic HCC but the combination with immune cell therapy is rarely reported (4–6). Chimeric antigen receptor (CAR) T-cell therapy has achieved outstanding efficacy in hematological malignancies and shown potential anti-tumor efficacy in early phase clinical trials for the treatments of solid tumors including advanced HCC (aHCC) (7, 8). Glypican-3 (GPC3) is an ideal immunotherapeutic target for HCC barely expressed in normal tissues and highly expressed in HCC (9, 10). In a previous report of two sequential phase 1 trials (NCT02395250 and NCT03146234) of glypican-3-targeted chimeric antigen receptor (CAR-GPC3) T-Cells (product code Y035) for aHCC patients, thirteen patients underwent CAR-GPC3 T cells with lymphodepletion regimens. Two out of 13 patients achieved partial response (PR) according to RECIST 1.1 (8). Based on its good safety profile and partial antitumor activities observed in the phase 1 trials, we explored a method to improve clinical response. Sorafenib is currently the recommended first-line systemic agent for aHCC but the ORR is just 2% to 4% (2, 11, 12). Sorafenib has been reported to enhance the efficacy of immunotherapeutic medications, therefore we explored the combination of CAR-GPC3 T cells with Sorafenib in mouse models of HCC (13, 14). The results demonstrated that via combination with Sorafenib, CAR-GPC3 T cells may be more effective against HCC than the CAR-T cells alone, probably by the mechanism of promoting IL-12 secretion of tumor-associated macrophages and tumor cell apoptosis in mouse models (15).

Here we report an aHCC patient who received the combination therapy of Sorafenib and CAR-GPC3 T-cell (product code CT011, former code Y035) in an investigator-initiated clinical trial (NCT03302403). The patient obtained PRs from the 3^rd^ month and achieved CR since the 12^th^ month post first infusion of CT011. The construction of CT011 CAR-GPC3 T cells was described previously and shown in **Supplementary Information** (8).

## Case Presentation

### The patient’s demography and prior treatment

**Figure 1:**
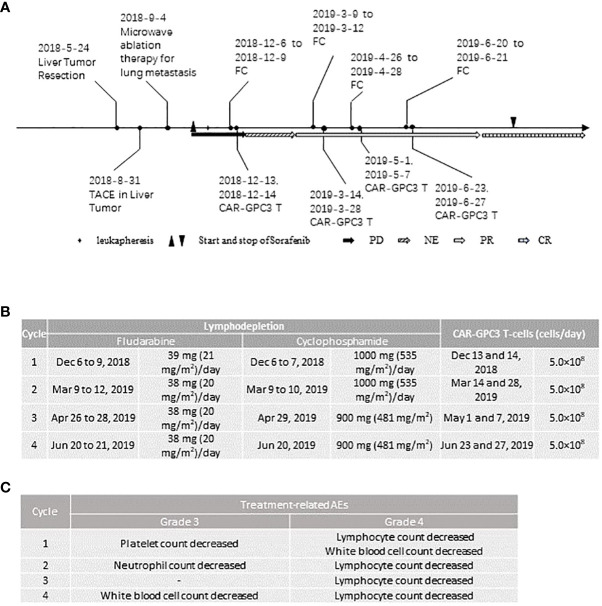
Clinical activities of the patient: **(A)**, Therapies and their responses; **(B)**, Dose of preconditioning treatment and CAR-GPC3 T-cells in each cycle; **(C)**, All the grade 3 and 4 treatment-related adverse events in each cycle. PD, progression disease; NE, not evaluable; PR, partial response; CR, complete response.

The patient was a 60-year-old Asian male. He was diagnosed with hepatitis B virus (HBV)-related HCC and underwent liver tumor resection in May 2018. The patient started to take entecavir 0.5mg QD continuously since Dec 2018 for HBV infection management. He was diagnosed with HCC recurrence and lung metastasis in August 2018. He then received trans-arterial chemo-embolization therapy for liver tumor on August 31, 2018, and microwave ablation therapy for lung metastasis on September 4, 2018. On November 6, 2018, the abdominal magnetic resonance imaging showed progressive disease (PD) (**Figure 1A**). He was enrolled in an investigator-initiated trial of “Clinical Study of Redirected Autologous T Cells with a Chimeric Antigen Receptor in Patients with Malignant Tumors” on November 10, 2018 (NCT03302403). The patient’s GPC-3 expression was ++ to +++ in 70% of tumor cells as detected by immunohistochemistry (**Supplementary Information**). The alpha-fetoprotein (AFP) was 6047 ng/ml at screening. There were four target lesions at baseline and the total diameter was 77.9 millimeter (mm): No.1 target was in the S6 segment of the liver (20.83 mm), No.2 target was next to the gallbladder fossa in the abdominal cavity (20.34 mm), No.3 target was at the right intra-abdominal cavity (16.76 mm), No.4 target was the lymph node in the mediastinum (19.97 mm) (**Supplementary Information**).

### Study Treatment

After completing informed consent and eligibility confirmation, the patient underwent leukapheresis to obtain peripheral blood mononuclear cells (PBMC) for the generation of CT011. CT011 were produced as described previously (8, 15). Seven days after leukapheresis, the patient started to receive Sorafenib of 400 mg twice daily. Sorafenib was administered during the CT011 treatment and lasted for 16 months post-first infusion. Prior to each cycle of CT011 infusion, the patient received preconditioning of cyclophosphamide 500 mg/m^2^/day and fludarabine 20-25 mg/m^2^/day. The details of the preconditioning regimen and CAR T infusion are shown in **Figure 1B**. Each cycle included two infusions of 5×10^8^ CAR-GPC3 T cells each time. The patient received 4 cycles of CAR-T therapy and 4×10^9^ CAR-GPC3 T cells in total were infused.

### Adverse Events (AEs)

All AEs were graded according to the Common Terminology Criteria for Adverse Events version 5.0. Cytokine release syndrome (CRS) and CAR-T-cell-related encephalopathy (CRES) events were assessed and graded per the Lee and CARPOX Working Group’s criteria (2014) (16, 17). The patient tolerated the combination therapy well. All the grade 3 or 4 AEs are shown in **Figure 1C**. The patient experienced treatment-related AE after each cycle of CT011 treatment. No treatment-related serious AE, neurotoxicity, or infusion reaction occurred. Most of the treatment-related AEs were grade 1 or 2, including CRS, anemia, chills, hypotension, pyrexia, hypoalbuminemia, hypokalemia, prolonged activated partial thromboplastin time, and so on. All the ≥ grade 3 AEs were expected hematological toxicities including decreased white blood cell count, lymphocytopenia, thrombocytopenia, and neutropenia, which were mainly due to preconditioning regimen and recovered after therapy within 2 weeks.

The CRS occurred after each cycle of CT011 treatment (Grade 2 in the first three cycles, and Grade 1 in the 4^th^ cycle. The main symptom was fever 24-48 hours after infusion. All the CRS were well managed by a single dose of tocilizumab (320 mg, 4 mg/kg).

### Persistence of CAR-GPC3 T cells and cytokines

**Figure 2:**
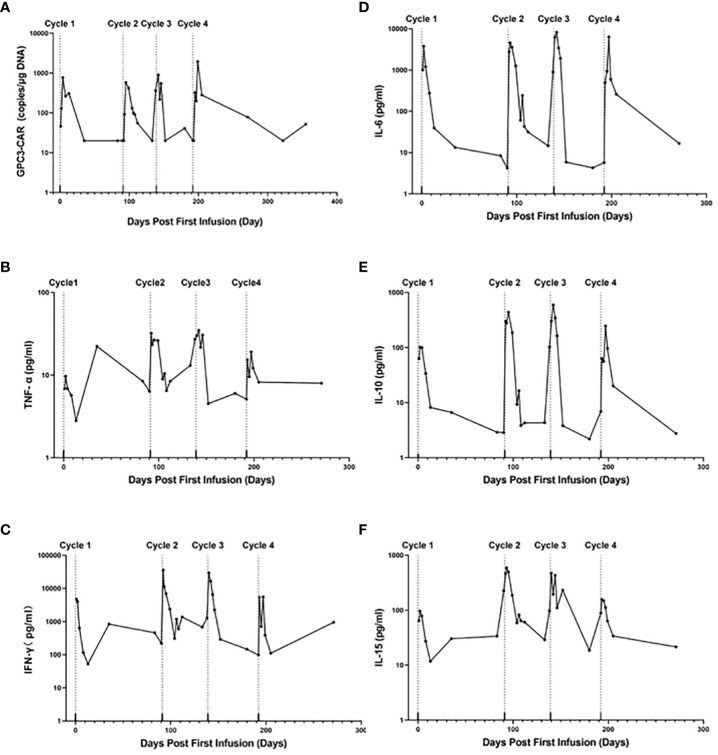
CAR-GPC3 expansion and cytokines in peripheral blood. **(A)**, The copies number of CAR-GPC3; **(B)**, TNF-a; **(C)**, IFN-γ; **(D)**, IL-6; **(E)**, IL-10; **(F)**, IL-15. The first cycle CAR T cells started on Day 0; the second cycle of CAR T cells started on Day 91; the third cycle of CAR T cells started on Day 139; The fourth cycle CAR T cells started on Day 192.

As shown in **Figure 2A**, the GPC3 CAR copies in peripheral blood rapidly increased to their peak at within one week post-infusion and then decreased gradually on each cycle. The highest peak number of CAR copies was reached on D7 post first infusion in the 4^th^ cycle of treatment (1945 copies/μg gDNA). As shown in **Figure 2 B–F**, cytokine tumor necrosis factor-126 α (TNF-α), interferon-γ (IFN-γ), interleukin-6 (IL-6), interleukin-10 (IL-10) and interleukin-15 (IL-15) were elevated and reached their peaks at 3 days after each cycle of CAR T-cell 127 infusion, and then dropped to normal level after about 2 weeks. and reached their peaks at 3 days after each cycle of CAR T-cell infusion, and then dropped to normal level after about 2 weeks.

### Anti-tumor activities (The AFP and tumor response evaluation)

**Figure 3:**
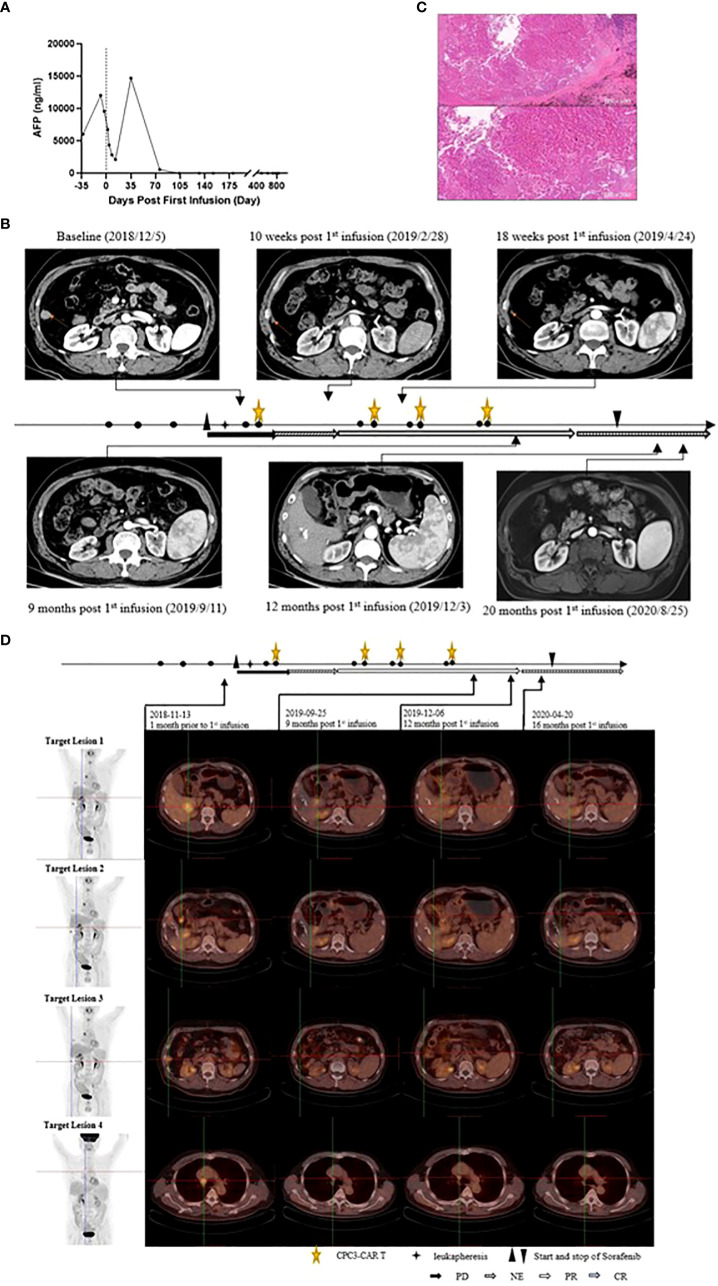
Efficacy of the combination of Sorafenib plus CT011 with lymphodepletion. **(A)**, Changes of the AFP after Sorafenib monotherapy and the initiation of GPC3-CAR T infusion; **(B)**, Changes of the No.3 target lesion. CT scans showed this target lesion was about 16.76 mm at baseline and regressed gradually after the combination treatment. At 9^th^ month, this lesion completely disappeared without relapse; **(C)**, The postoperative pathology picture of the No.4 target lesion at 12th month (HE×100 and HE×200, the pictures showed large patch of coagulative necrosis, peripheral fibrosis, chronic inflammatory cell infiltration, no obvious tumor residue, indicating reaction post anti-tumor treatment); **(D)**, Positron emission tomography-computed tomography (PET-CT) images of the target lesions. The patient received PET-CT check at baseline, 9 months, 12 months, and 16 months post first infusion. No.1, No.2 and No.3 target lesions disappeared at the 12^th^ month, while No.4 target lesion was inactive and was considered as non-specific uptake after anti-tumor treatment.

As shown in **Figure 3A** and **Supplementary Information**, before preconditioning, the AFP increased to 12,049 ng/mL from 6,047 ng/mL at the screening when the patient had received Sorafenib therapy for two weeks. After 13 days of the first infusion of CT011, the AFP declined to 2,104.55 ng/mL. Interestingly, the AFP was abnormally increased up to 14,714 ng/mL on the 35^th^ day and decreased to 557 ng/mL on the 73^rd^ day. The AFP level continuously declined to the normal value (≤9 ng/mL) after the first infusion of the second cycle and remained normal afterwards.

According to RECIST 1.1, the tumor response evaluation was PR at the 3^rd^ month following the first cycle of CT011 and then four PRs were obtained in the following approximately 8 months. The tumor response was then converted to CR at the 12^th^ month, as was confirmed by positron emission tomography-computed tomography (PET-CT) (**Figures 1A** and **3**). The CT images at scheduled visits post baseline in the 9^th^ month after first infusion are shown in **Figure 3B** and **Supplementary Information**. In the 12^th^ month after the first infusion of CT011, three of four target lesions disappeared except for the No. 4 target lesion (mediastinal lymph node that stabilized at about 10 mm afterwards. The patient also underwent a thoracoscopic mediastinal lymph node resection in the 12^th^ month, and the postoperative pathology indicated no tumor cell residue, suggesting a reaction to CT011 and Sorafenib combination treatment (shown in **Figure 3C**). **Figure 3D** show PET-CT images at baseline, 9 months, 12 months, and 16 months post first infusion. No. 1, No. 2, and No. 3 target lesions disappeared, and the No.4 target lesion was considered inactive with a non-specific uptake after anti-tumor treatment (**Supplementary Information**).

## Discussion/Conclusion

The efficacy of CAR T cells against solid tumors remains unsatisfactory due to barriers including heterogeneous tumor antigen expression, an immunosuppressive and hostile tumor microenvironment, insufficient infiltration into the tumor sites and poor CAR T persistence (18). To overcome those challenges and enhance the efficacy, the combination of CAR T-cell with other anticancer therapies is being developed (19, 20).

GPC3 is specifically overexpressed in more than 50%-70% of HCCs, making it an ideal treatment target for HCC (21, 22). GPC3 positive HCC patients had a significantly lower 5-year disease-free survival rate (27% vs 62%, p = 0.0036) and five-year survival rate (54.5% vs 87.7%, p = 0.031) than GPC3-negative patients (23, 24). The expression of GPC3 is an independent prognostic factor of recurrence after hepatectomy (23). In recent years, multiple GPC3 targeted therapies have been investigated, including GPC3 antibodies, GPC3-derived vaccines, and immunotoxins, but very limited positive progress in clinical trials has been reported, especially for aHCC (25, 26). The safety and anti-tumor efficacy of various GPC3-targeting CAR T therapies in HCCs were confirmed in preclinical studies (15, 27–30). While further clinical trial investigation is required to verify the safety and anti-tumor efficacy in patients, CAR-GPC3 T therapy is recognized as a potential therapy for HCC (27–30). Our phase 1 clinical trials (NCT02395250 and NCT03146234) of GPC3 CAR T with lymphodepletion shows good tolerability in refractory or relapsed GPC3 positive HCCs with promising potential antitumor activities that need further improvement (31).

Sorafenib is recommended as a first-line oral systemic therapy for HCC which has been reported to have immune-modulatory effects (32–34). It can modulate the cytokine phenotype of macrophage toward a profile that promotes the function of immune effector cells. It was reported that Sorafenib reverse the suppression of IL-12 stimulated with lipopolysaccharide and/or prostaglandin E_2_ (15, 35, 36). Sorafenib can also reverse immunosuppression by decreasing and inhibiting the accumulation of myeloid-derived suppressor cells and immature dendritic cells in the tumor microenvironment or directly through the vascular endothelial growth factor (VEGF) or VEGF receptor (VEGFR) pathway inhibition on Treg (37). Furthermore, Sorafenib can also promote vascular normalization, a process of tumor microenvironment remolding, realizing the transition from immune suppression to immune support (38). In our previous preclinical studies, we demonstrated that Sorafenib could change the tumor microenvironment and enhance the antitumor activities of CAR-GPC3 T-cells against HCC (15). Thus, we explored the combination therapy in clinical practice.

The patient in this report received the CAR-GPC3 T-cells and Sorafenib combination treatment. He obtained PRs from the 3^rd^ month and achieved CR in the 12^th^ month after his first infusion. As of December 7, 2021, no progression has been identified for more than 36 months. For this patient, the AFP level increased and his disease was progressing when receiving Sorafenib alone before lymphodepletion. This AFP level was significantly decreased to normal level after CT011 infusion, although there was ever a transient elevation of D35 post first infusion, which could likely be ascribed to tumor cell necrosis and its promotion of the release of large amounts of tumor antigens (39, 40). The AFP level reduced to the normal range after the second cycle of CT011 infusion and has remained normal to the present, in line with tumor remissions observed via imaging scan.

After each infusion, in the presence of Sorafenib the peak values of CAR-GPC3 copies reached 700, 580, 900 and 1945 copies/μg gDNA, respectively. The patient partially responded to the combination treatment and later gradually achieved CR after 4 cycles of CT011 infusions, which warranted further investigation to determine the additional value of multiple infusions and the combination therapy to achieve superior efficacy of CAR T-cell in a patient with HCC.

Consistent with good safety profile in the phase 1 study, the patient was well tolerated to the CAR-GPC3 T multiple infusions in combination with Sorafenib (8). After each infusion, the CAR-GPC3 copies and serum levels of cytokines increased significantly in the presence of Sorafenib. The occurrence and severity of AEs in additional cycles were slightly lower than those in the first cycle, which may be due to the reduced tumor burden, an impacting factor in CRS severity.

In summary, we report the first patient treated with CAR-GPC3 T-cell and Sorafenib combination therapy. To the best of our knowledge, this is the first reported case with a CR after the combination therapy of CAR-T cells with tyrosine kinase inhibitors. The clinical outcome demonstrated that the combination therapy of CAR-GPC3 T-cell and Sorafenib may be a new promising approach for GPC3+ aHCC patients. A well-designed study is deemed to be necessary to further confirm the safety and efficacy of the combination therapy.

## Data availability statement

The original contributions presented in the report are included in the article/**Supplementary Material**. Further inquiries can be directed to the corresponding author.

## Ethics statement

The studies involving human participants were reviewed and approved by ethics committee of The First Affiliated Hospital of Wenzhou Medical University. Written informed consent was obtained from participants for publication of the details of their medical case and any accompanying images.

## Author contributions

The manuscript was reviewed and revised by all the authors. All authors contributed to the analysis and interpretation of the data. The authors affirm the accuracy and completeness of the data and adherence of the study to the protocol.

## Funding

This study was funded by CARsgen Therapeutics Co., Ltd.

## Conflict of interest

Authors ZW, JX, HW, WW, and ZL are employed by CARsgen Therapeutics Co., Ltd.

The remaining authors declare that the research was conducted in the absence of any commercial or financial relationships that could be construed as a potential conflict of interest.

This study received funding from CARsgen Therapeutics Co., Ltd. The funder provided the manufactory of CT011 product and analysis of biological samples. The funder had also involved with the study design, review, and revision of the case report.

## Publisher’s note

All claims expressed in this article are solely those of the authors and do not necessarily represent those of their affiliated organizations, or those of the publisher, the editors and the reviewers. Any product that may be evaluated in this article, or claim that may be made by its manufacturer, is not guaranteed or endorsed by the publisher.
