# Supplementary material for: Long term complete response of advanced hepatocellular carcinoma to glypican-3 specific chimeric antigen receptor T-Cells plus sorafenib, a case report
Source: Front Immunol. 2022 Aug 17;13:963031. doi: 10.3389/fimmu.2022.963031 (PMC9428446; doi:10.3389/fimmu.2022.963031)
Supplement: Supplementary file 1 [file DataSheet_1.pdf]

Supplementary Materials

Supplementary Figure 1 CAR-GPC3 construct 2
Supplementary Figure 2 GPC3 expression..3
Supplementary Figure 3 CT images of Target Lesions 4
Supplementary Table 1. Change of Target Lesions Size 6
Supplementary Table 2. Number of AFP level 8
Supplementary Table 3. Number of Cytokines..9
Supplementary Table 4. All the Adverse Events 10

Supplementary Figure 1 CAR-GPC3 construct

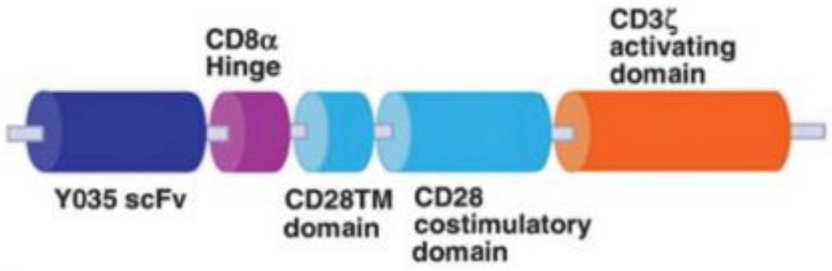

The modular composition of CT011 (former name Y035) CAR-GPC3. scFv, single-chain variable fragment.

CT011 consisted of a humanized anti-GPC3 single-chain variable fragment, CD8α hinge domain, CD8α transmembrane domain, CD28 intracellular domain, and CD3ζ intracellular signaling domain that were cloned into a lentiviral backbone.

**This figure has been published on Chimeric Antigen Receptor-Glypican-3 T-Cell Therapy for Advanced Hepatocellular Carcinoma: Results of Phase I Trials. Clinical cancer research : an official journal of the American Association for Cancer Research. 2020 Aug 1;26(15):3979-89. PubMed PMID: 32371538. Epub 2020/05/07. eng.*
https://pubmed.ncbi.nlm.nih.gov/32371538/

Supplementary Figure 2 GPC3 expression

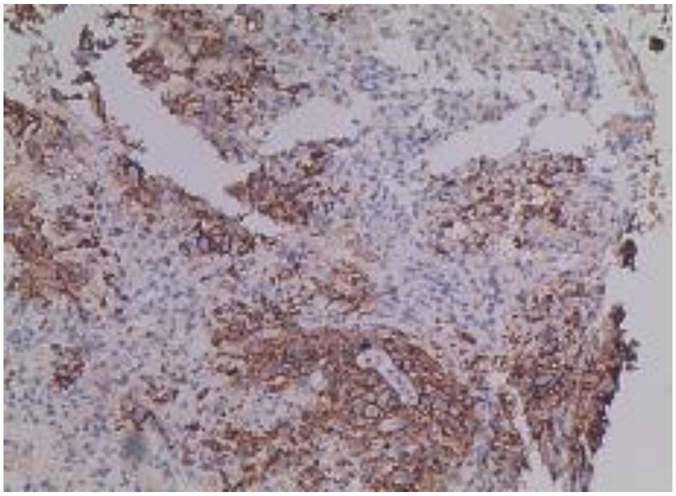

GPC3 expression of the patient was detected by immunohistochemistry. The expression intensity of GPC3 was ++ to +++, and the expression rate was 70%.

Supplementary Figure 3 CT images of Target Lesions

Target Lesion No 1 - the S6 segment of the liver

Baseline (2018/12/5)	10 weeks post 1st infusion (2019/2/28)
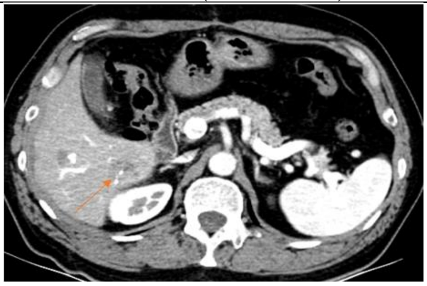	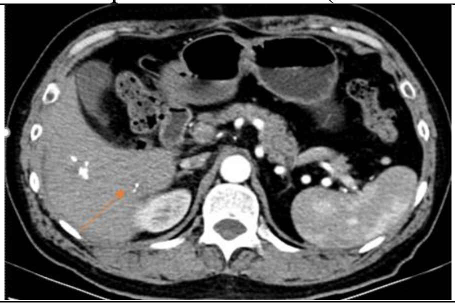
18 weeks post 1st infusion (2019/4/24)	9 months post 1st infusion (2019/9/11)
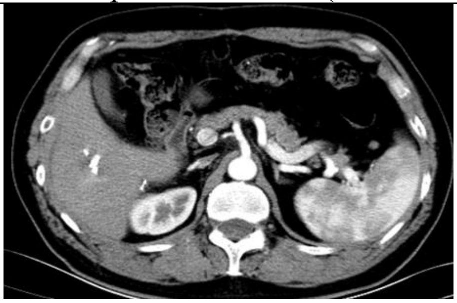	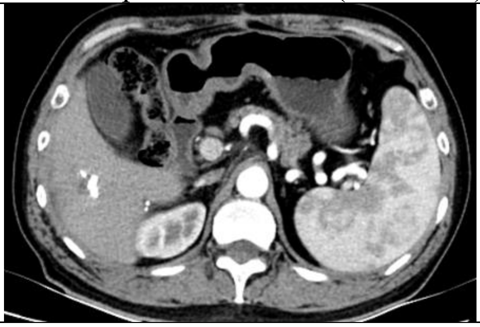
12 months post 1st infusion (2019/12/3)	20 months post 1st infusion (2020/8/25)
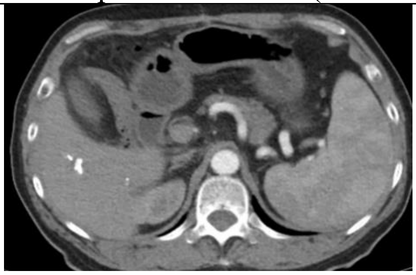	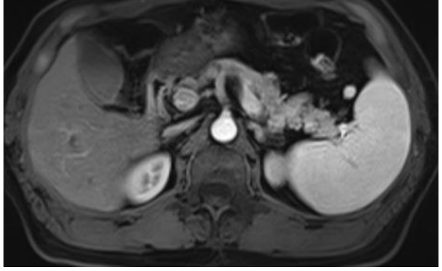

Target Lesion No 2 - next to the gallbladder fossa in the abdominal cavity

Baseline (2018/12/5)	10 weeks post 1st infusion (2019/2/28)
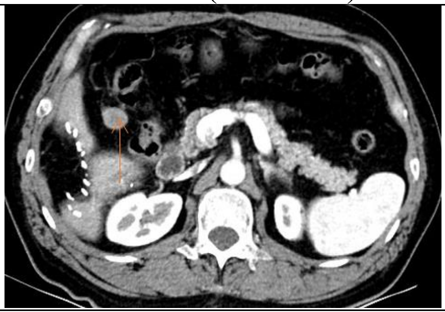	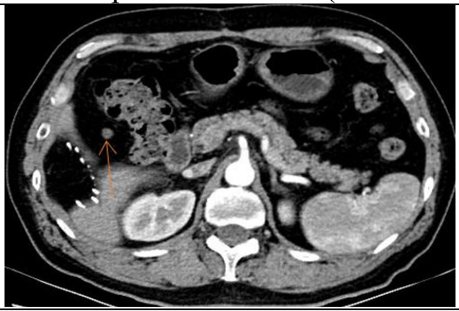
18 weeks post 1st infusion (2019/4/24)	9 months post 1st infusion (2019/9/11)

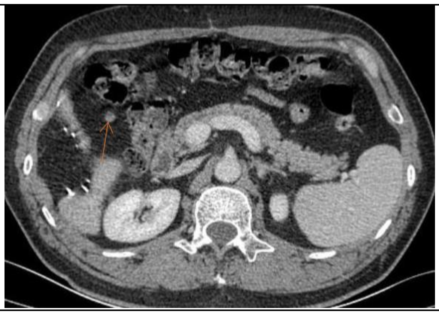	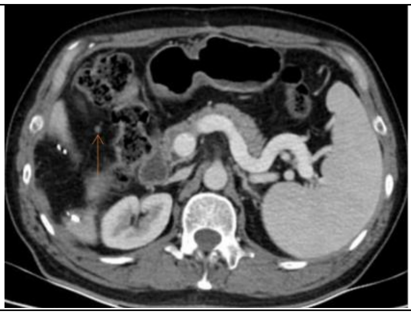
12 months post 1st infusion (2019/12/3)	20 months post 1st infusion (2020/8/25)
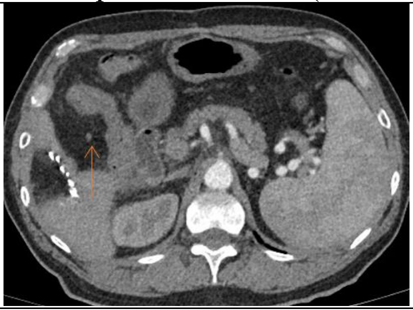	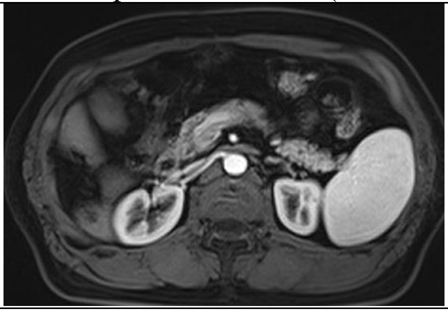
**Target Lesion No 4 - the Lymph node in the mediastinum**	
Baseline (2018/11/13)	18 weeks post 1st infusion (2019/4/24)
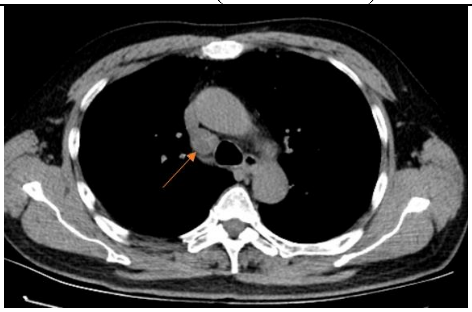	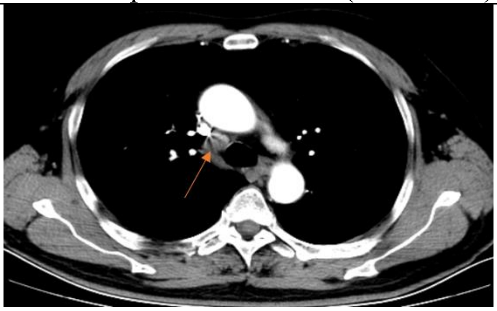
9 months post 1st infusion (2019/9/11)	12 months post 1st infusion (2019/12/3)
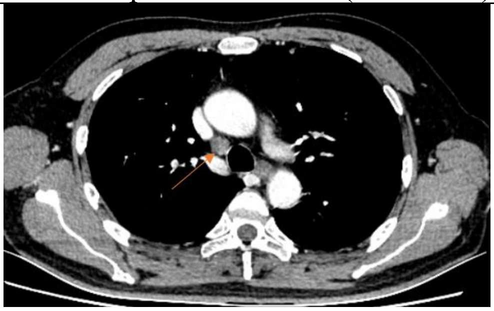	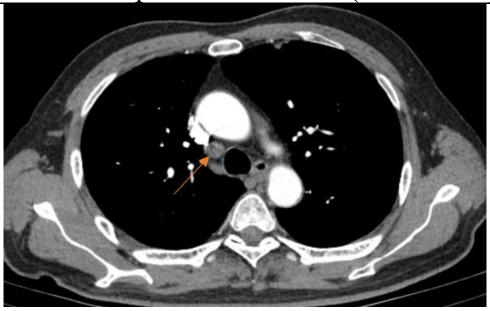

CT scans showed No. 1,2 and 3 target lesions completely disappeared, and No. 4 target lesions was considered inactive after CAR-GPC3 T infusion.

Supplementary Table 1. Change of Target Lesions Size

Date	Organ	No.	Method	Location of Lesions	Size (mm)
2018/12/5	Liver	T1	CT	the S6 segment of the liver	20.83
2019/1/17	Liver	T1	CT	the S6 segment of the liver	12.67
2019/2/28	Liver	T1	CT	the S6 segment of the liver	6.02
2019/4/24	Liver	T1	CT	the S6 segment of the liver	0
2019/6/11	Liver	T1	CT	the S6 segment of the liver	0
2019/9/11	Liver	T1	Contrast-enhanced CT	the S6 segment of the liver	0
2019/10/29	Liver	T1	Contrast-enhanced CT	the S6 segment of the liver	0
2019/12/3	Liver	T1	CT	the S6 segment of the liver	0
2020/4/20	Liver	T1	PET CT	the S6 segment of the liver	0
2020/4/20	Liver	T1	PET CT	the S6 segment of the liver	0
2020/8/25	Liver	T1	MRI	the S6 segment of the liver	0
2021/3/24	Liver	T1	MRI	the S6 segment of the liver	0
2018/12/5	Gallbladder	T2	CT	next to the gallbladder fossa in the abdominal cavity	20.34
2019/1/17	Gallbladder	T2	CT	next to the gallbladder fossa in the abdominal cavity	8.1
2019/2/28	Gallbladder	T2	CT	next to the gallbladder fossa in the abdominal cavity	8.45
2019/4/24	Gallbladder	T2	CT	next to the gallbladder fossa in the abdominal cavity	5
2019/6/11	Gallbladder	T2	CT	next to the gallbladder fossa in the abdominal cavity	3.95
2019/9/11	Gallbladder	T2	Contrast-enhanced CT	next to the gallbladder fossa in the abdominal cavity	4.10
2019/10/29	Gallbladder	T2	Contrast-enhanced CT	next to the gallbladder fossa in the abdominal cavity	3.52
2019/12/3	Gallbladder	T2	CT	next to the gallbladder fossa in the abdominal cavity	3.09
2020/4/20	Gallbladder	T2	PET CT	next to the gallbladder fossa in the abdominal cavity	0
2020/8/25	Gallbladder	T2	MRI	next to the gallbladder fossa in the abdominal cavity	0
2021/3/24	Gallbladder	T2	MRI	next to the gallbladder fossa in the abdominal cavity	0

Date	Organ	No.	Method	Location of Lesions	Size (mm)
2018/12/5	abdominal cavity	T3	CT	at the right intra-abdominal cavity	16.76
2019/1/17	abdominal cavity	T3	CT	at the right intra-abdominal cavity	7.99
2019/2/28	abdominal cavity	T3	CT	at the right intra-abdominal cavity	7.3
2019/4/24	abdominal cavity	T3	CT	at the right intra-abdominal cavity	4.74
2019/6/11	abdominal cavity	T3	CT	at the right intra-abdominal cavity	0
2019/9/11	abdominal cavity	T3	Contrast-enhanced CT	at the right intra-abdominal cavity	0
2019/10/29	abdominal cavity	T3	Contrast-enhanced CT	at the right intra-abdominal cavity	0
2019/12/3	abdominal cavity	T3	CT	at the right intra-abdominal cavity	0
2019/12/6	abdominal cavity	T3	PET CT	at the right intra-abdominal cavity	0
2020/4/20	abdominal cavity	T3	PET CT	at the right intra-abdominal cavity	0
2020/8/25	abdominal cavity	T3	MRI	at the right intra-abdominal cavity	0
2021/3/24	abdominal cavity	T3	MRI	at the right intra-abdominal cavity	0
2018/11/3	the Lymph node	T4	CT	the Lymph node in the mediastinum	19.97
2019/3/2	the Lymph node	T4	CT	the Lymph node in the mediastinum	20.31
2019/4/24	the Lymph node	T4	CT	the Lymph node in the mediastinum	16.8
2019/6/11	the Lymph node	T4	CT	the Lymph node in the mediastinum	14.14
2019/9/11	the Lymph node	T4	Contrast-enhanced CT	the Lymph node in the mediastinum	13.01
2019/10/29	the Lymph node	T4	Contrast-enhanced CT	the Lymph node in the mediastinum	12.13
2019/12/3	the Lymph node	T4	Contrast-enhanced CT	the Lymph node in the mediastinum	12.52
2019/12/6	the Lymph node	T4	PET CT	the Lymph node in the mediastinum	0
2020/4/20	the Lymph node	T4	PET CT	the Lymph node in the mediastinum	0
2020/8/24	the Lymph node	T4	MRI	the Lymph node in the mediastinum	0
2021/3/24	the Lymph node	T4	MRI	the Lymph node in the mediastinum	0

Supplementary Table 2. Number of AFP level

Date	Days Post First Infusion (Day)	AFP(ng/ml)
2018/11/10	-33	6047
2018/12/5	-8	12046
2018/12/10	-3	9579
2018/12/15	2	6751
2018/12/17	4	4363
2018/12/21	8	2822
2018/12/26	13	2105
2019/1/17	35	14714
2019/2/27	76	557
2019/3/27	104	9
2019/4/24	132	2
2019/5/14	152	3
2019/6/11	180	1
2019/7/6	205	3
2019/9/10	271	1
2019/10/28	319	1
2019/12/3	355	2
2020/4/20	494	1
2020/8/24	620	1
2020/12/6	724	2
2021/1/31	780	1
2021/3/24	832	3

Supplementary Table 3. Number of Cytokines

Days after 1st CART infusion (d)	IFN-γ （pg/ml）	IL-10 （pg/ml）	IL-15 （pg/ml）	IL-6 （pg/ml）	TNF-α （pg/ml）
-9	10.38	0.27	2.43	0.44	0.95
-4	10.35	0.28	29.63	1.01	1.12
1	4728.07	62.94	64.64	1003.19	6.90
2	4018.38	101.80	96.46	3778.08	9.71
4	643.61	99.78	78.37	1197.95	6.87
8	116.77	33.97	27.43	275.54	5.70
13	52.85	8.17	11.72	39.51	2.81
35	845.30	6.64	30.40	13.36	22.30
83	465.61	2.90	33.87	8.41	8.45
90	221.40	2.86	225.39	4.21	6.37
92	36180.20	301.70	461.49	2794.94	32.01
93	11397.42	279.59	588.30	4591.11	23.38
95	6968.09	437.10	498.38	3551.88	26.64
99	2401.60	187.29	186.88	1264.15	26.19
104	314.58	9.27	59.04	61.10	8.93
106	1182.91	16.49	82.38	242.05	10.46
108	605.49	3.84	64.54	43.01	6.48
112	1376.99	4.28	60.49	31.45	8.45
133	685.80	4.33	29.03	14.63	13.12
138	1286.18	101.85	96.96	900.04	27.25
140	29924.91	303.61	470.92	6262.35	29.92
142	16535.38	597.56	194.34	8297.72	34.94
144	6564.22	344.71	430.46	3428.67	21.69
146	2243.30	162.88	111.07	1922.83	30.55
152	292.93	3.81	230.89	5.88	4.51
180	145.81	2.18	18.61	4.29	6.01
192	98.60	6.93	88.79	5.70	5.12
193	5397.09	63.30	156.36	499.37	15.37
195	716.75	56.12	149.25	937.34	9.56
197	5585.43	245.72	112.31	6353.13	19.09
199	389.72	96.50	63.76	596.17	12.15
205	110.71	20.10	34.05	259.37	8.23
271	958.92	2.75	21.49	16.61	7.98

Supplementary Table 4. All the Adverse Events

Adverse Events	CTCAE grade	relationship with preconditioning	relationship with CAR T cells
Lymphocytopenia	4	Definitely Related	Unrelated
White blood cell count decreased	4	Related	Possibly unrelated
Thrombocytopenia	3	Possibly Related	Possibly Related
Hypokalemia	1	Possibly Related	Unrelated
Hypoalbuminemia	2	Possibly Related	Possibly Related
Pyrexia	2	Possibly unrelated	Related
Weakness	1	Possibly unrelated	Possibly Related
Chills	1	Possibly unrelated	Related
Chest discomfort	1	Unrelated	Possibly Related
Sore throat discomfort,	1	Possibly unrelated	Possibly Related
Cytokine release syndrome	2	Unrelated	Related
Dyspnea	1	Possibly unrelated	Possibly Related
The time of activated partial thromboplastin prolonged	1	Possibly unrelated	Possibly Related
Hypocalcemia	1	Possibly Related	Possibly Related
Expectoration	1	Possibly unrelated	Possibly Related
Total bilirubin increased	1	Possibly Related	Unrelated
Rash	1	Possibly unrelated	Possibly Related
Cytokine release syndrome	2	Unrelated	Related
Thrombocytopenia	1	Possibly Related	Unrelated
Anemia	1	Possibly Related	Possibly Related
Lymphocytopenia	4	Related	Possibly Related
The time of activated partial Thromboplastin prolonged	1	Possibly unrelated	Possibly Related
Hypoalbuminemia	1	Possibly unrelated	Possibly Related
Hypokalemia	2	Unrelated	Possibly Related
Hypokalemia	2	Unrelated	Possibly Related
Hypoalbuminemia	1	Possibly unrelated	Possibly Related
Cytokine release syndrome	1	Unrelated	Related
Cytokine release syndrome	2	Unrelated	Related
Pyrexia	2	Unrelated	Related
Pyrexia	2	Unrelated	Related
The time of activated partial thromboplastin prolonged	1	Possibly unrelated	Possibly Related
Neutrophil count decreased	1	Possibly Related	Possibly Related
The time of activated partial thromboplastin prolonged	1	Possibly unrelated	Possibly Related
Neutropenia	1	Possibly Related	Possibly Related
Neutropenia	3	Possibly Related	Possibly Related
Neutropenia	1	Possibly Related	Possibly Related
Hypokalemia	2	Possibly unrelated	Possibly Related
Hypokalemia	2	Possibly Related	Possibly Related

Adverse Events	CTCAE grade	relationship with preconditioning	relationship with CAR T cells
Hypokalemia	2	Possibly Related	Possibly Related
Thrombocytopenia	2	Possibly Related	Possibly Related
Thrombocytopenia	1	Possibly Related	Possibly Related
Anemia	1	Possibly Related	Possibly Related
Anemia	1	Possibly Related	Possibly Related
Hypoalbuminemia	1	Possibly unrelated	Possibly Related
Hypoalbuminemia	1	Possibly unrelated	Possibly Related
Chills	1	Unrelated	Possibly Related
Hypotension	1	Unrelated	Possibly Related
Pyrexia	2	Unrelated	Possibly Related
Hypotension	1	Possibly unrelated	Possibly Related
Hypotension	1	Possibly unrelated	Possibly Related
Tachycardia	1	Possibly unrelated	Possibly Related
Cough	1	Possibly unrelated	Possibly Related
Leukopenia	1	Related	Possibly unrelated
Leukopenia	1	Related	Possibly unrelated
Leukopenia	2	Related	Possibly unrelated
Leukopenia	2	Related	Possibly unrelated
Leukopenia	3	Related	Possibly unrelated
Hypocalcemia	1	Possibly unrelated	Possibly Related
Hypocalcemia	1	Possibly unrelated	Possibly Related
